# Neither endothelial function nor carotid artery intima-media thickness predicts coronary computed tomography angiography plaque burden in clinically healthy subjects: a cross-sectional study

**DOI:** 10.1186/s12872-015-0061-x

**Published:** 2015-07-07

**Authors:** Elin B. Brolin, Stefan Agewall, Torkel B. Brismar, Kenneth Caidahl, Per Tornvall, Kerstin Cederlund

**Affiliations:** Department of Radiology, Karolinska University Hospital Huddinge, Stockholm, 141 86 Sweden; Department of Clinical Science, Intervention and Technology, Division of Medical Imaging and Technology at Karolinska Institutet, Stockholm, 141 86 Sweden; Department of Cardiology, Oslo University Hospital Ullevål; Institute of Clinical Sciences, University of Oslo, Oslo, Norway; Department of Clinical Physiology, Karolinska University Hospital, Stockholm, Sweden; Department of Molecular Medicine and Surgery, Karolinska Institutet, Stockholm, Sweden; Institution for Clinical Science and Education at Södersjukhuset, Karolinska Institutet, Stockholm, Sweden

**Keywords:** CAD, Coronary artery disease, RH-PAT, PAT, Endothelial function, IMT, CIMT, Intima-media thickness, Coronary CTA, Coronary computed tomography angiography

## Abstract

**Background:**

Cardiovascular risk assessment is usually based on traditional risk factors and risk assessment algorithms. However, a number of risk markers that might provide additional predictive power have been identified. Endothelial function determined by digital reactive hyperemia peripheral arterial tonometry (RH-PAT) and carotid artery intima-media thickness (IMT) have both been proposed as surrogate markers for coronary artery disease (CAD). We aimed to examine the ability of RH-PAT and IMT to predict coronary computed tomography angiography (CTA) plaque burden in clinically healthy subjects.

**Methods:**

Fifty-eight clinically healthy volunteers (50–73 years old) underwent testing for RH-PAT and IMT as well as coronary CTA, including coronary artery calcium (CAC) scoring. Coronary CTA was analyzed with respect to any atheromatous plaques, stenotic as well as non-stenotic. The Mann–Whitney U-test was used to compare the groups with and without CAD and the Spearman test was used to test for correlation between variables.

**Results:**

Twenty-five (43 %) subjects had normal coronary arteries, without any signs of atherosclerosis. The median (range) number of diseased segments was 1 (0–10), RH-PAT index 2.2 (1.4-4.9), IMT 0.70 (0.49-0.99) mm and CAC 4 (0–1882). There was no association between presence or extent of CAD and RH-PAT index (Spearman correlation coefficient r_s_ = 0.13) or IMT (r_s_ = 0.098). As expected, CAC was strongly correlated to presence and extent of CAD by coronary CTA (r_s_ =0.86; p < 0.0001).

**Conclusions:**

Neither evaluation of endothelial function by RH-PAT nor assessment of carotid artery IMT can reliably be used to predict coronary CTA plaque burden in clinically healthy subjects.

## Background

Cardiovascular risk assessment, which is of great importance for guiding early preventive interventions and treatment, is usually based on traditional risk factors and risk assessment algorithms, such as the Framingham, the SCORE or the Reynolds risk scores [1–3]. In the search for easily accessible non-invasive tests that might provide additional predictive power, a number of novel risk markers have been identified [4].

Endothelial dysfunction is an early marker of atherothrombotic disease, being closely related to cardiovascular risk factors and also predicting cardiovascular events in patients with or without established cardiovascular disease [5, 6]. Since endothelial dysfunction is a systemic disorder, measurement of peripheral endothelial function can be used as a surrogate for coronary endothelial function [7, 8]. A quite recently developed method for assessing peripheral endothelial function is measuring digital reactive hyperemia peripheral arterial tonometry (RH-PAT), which has the advantage of being an easily accessible, largely operator-independent and highly reproducible method [9, 10]. A recent study demonstrated an association between endothelial function determined by RH-PAT and several cardio-metabolic risk factors, in particular male sex, overweight, smoking and low levels of HDL cholesterol [11].

A well established non-invasive surrogate marker for CAD is ultrasound measurement of the carotid artery intima-media thickness (IMT), which has proven to be associated with prevalent cardiovascular disease and to predict cardiovascular events [12, 13]. More recent studies however have challenged this role of IMT [14].

Numerous studies have shown that coronary artery calcium (CAC) scoring using cardiac computed tomography (CT) improves prediction of coronary heart disease events beyond traditional risk factors and risk scores [4, 15].

Coronary CT angiography (CTA) has evolved substantially during the last few years, and has been shown to entail very good accuracy when it comes to diagnosing stenoses of the coronary arteries as compared to conventional coronary angiography [16]. In addition to showing the lumen of the coronary arteries, coronary CTA also permits visualization of the vessel wall and thus detection of atherosclerotic plaques even when they do not give rise to stenoses. Accordingly, coronary CTA has also been shown to be highly sensitive in detecting atherosclerotic plaques of the coronary arteries when compared to intravascular ultrasound [17]. A number of recent studies have demonstrated a prognostic value of coronary CTA for risk prediction of adverse cardiovascular events in symtomatic and asymtomatic patients [18–20].

Even though measurements of peripheral endothelial function and IMT are supposed to reflect the presence and extent of CAD, only a few studies have compared these measures with plaque burden of the coronary arteries, as determined by coronary CTA [21]. Even fewer have focused on clinically healthy subjects, with predominantly low to intermediate risk profiles [22].

To clarify whether these non-invasive tests can reliably be used to predict coronary plaque burden in clinically healthy subjects, we studied the correlation of endothelial function and IMT respectively with plaque burden, as assessed by coronary CTA. For comparison we also studied the ability of CAC to predict plaque burden in the same group.

## Methods

The study conforms to the principles of the Declaration of Helsinki and was approved by the Regional Ethical Review Board in Stockholm and by the Radiation Protection Committee of the Karolinska University Hospital. Written informed consent was obtained from all study participants.

### Study group

The study group consisted of fifty-eight healthy volunteers between 50 and 73 years old, who were recruited in the Stockholm metropolitan area between June 2007 and May 2011, through a registry comprising all Stockholm residents. If they were willing to participate and had no previously known cardiovascular disease they underwent an exercise stress test, and if the test was normal they were invited to take part in the study. Exclusion criteria were previous adverse reaction to iodinated contrast media, renal dysfunction and arrhythmia (jeopardizing the diagnostic quality of the CT scan).

### Endothelial function assessment

Endothelial function was assessed using the digital reactive hyperemia peripheral arterial tonometry (RH-PAT) device EndoPAT (Itamar Medical Ltd., Caesarea, Israel), according to the method described in detail by Hamburg et al [23]. The method involves measuring pulse wave amplitude of the fingertip (namely the PAT signal), by use of a fingertip probe, at rest and after a 5-minute occlusion of the brachial artery, using a standard blood pressure cuff inflated to supra-systolic pressure. When the cuff is deflated, the surge of blood causes an endothelium-dependent flow mediated dilatation leading to reactive hyperemia and an increase in the PAT signal amplitude. A fingertip probe is also placed on the index finger of the contra lateral side for internal control. As a measure of reactive hyperemia the reactive hyperemia index (RHI) was calculated, using dedicated software, as the ratio between the post-occlusion and the pre-occlusion PAT signal amplitude divided by the corresponding ratio of the contra lateral side, to adjust for any spontaneous or systemic alterations of vascular tone. The test was performed in a thermo-neutral and quiet surrounding, after fasting and avoiding pre-test smoking/ snuff or consumption of caffeine.

### Carotid artery ultrasound

Two-dimensional images of the left and right common carotid artery (CCA) were acquired using an ultrasound scanner equipped with a 12 MHz transducer (Vivid 7, General Electric Company, Horten, Norway). From each CCA a long-axis cine loop of three beats and three diastolic images at the time of the electrocardiographic R-wave were digitally stored for off-line analysis. The intima-media thickness (IMT) of the CCA far wall was measured in three diastolic images using semi-automatic IMT analysis software (General Electric Company). A 10 mm region of interest (ROI) was manually placed starting 1 cm proximal to the carotid bulb. The borders between the intima-media of the far wall and the lumen or the adventitia respectively, were identified automatically. In case of suboptimal tracking, the ROI was adjusted or another diastolic frame was chosen. Manual correction was not performed. The left and right IMT results were calculated as the mean of three semi-automatic measurements, and finally the average of these two results was obtained. All examinations were performed and interpreted by the same experienced ultrasonographer, who did not have access to the coronary CTA findings.

### Coronary CTA data acquisition and analysis

Examinations were performed on a 64-slice CT scanner (LightSpeed VCT XT; GE Healthcare, Milwaukee, WI, USA). A prospectively ECG-triggered scan protocol was used: detector configuration 64 x 0.625 mm, rotation time 350 ms, tube potential 120 kV, tube current 450–650 mA (according to patient size), padding 0–200 ms (depending on heart rate variability). The contrast agent used was iodixanol 320 mg I/ml (Visipaque, GE Healthcare, Stockholm, Sweden), which was administered using a dual-head injector (Medrad, Stellant Dual Head Injector, Pittsburgh, PA, USA) and a triple-phase protocol. The contrast agent was individually dosed, based on body weight (400 mg I/kg, 75–100 ml iodixanol) with a fixed injection time (15 s), resulting in an injection rate of 5–7 ml/s. This was followed by a 50 ml mixture of 40 % iodixanol and 60 % saline and finally by a 50 ml saline chaser. Bolus tracking software was used (SmartPrep, GE Healthcare, Milwaukee, WI, USA) in order to synchronize image acquisition with optimal contrast opacification of the coronary arteries. In the absence of contraindications and depending on the initial heart rate, patients received metoprolol (25–100 mg) per os prior to the examination. Patients also received sublingual nitroglycerine (0.4 mg) 4 minutes before the scan.

The coronary CTA exam was analyzed independently by three readers (two experienced readers with level 2 and one reader with level 1 according to ACCF/AHA levels of competence) [24], who were blinded to all clinical information as well as to the results of endothelial function and IMT testing. A subsequent joint reading was performed and a consensus reached. Coronary CTA data analysis was performed using the CardIQ Xpress software on the Advantage Workstation 4.4 (GE Healthcare, Milwaukee, WI, USA). Axial source images, multiplanar and curved multiplanar reformats as well as thin-slab maximum intensity projections were used. The optimal image display setting for lumen and plaque assessment was chosen on an individual basis, but in general at a window width of 800–1000 HU and a level of 100–200 HU. Coronary arteries were subdivided into 17 segments, according to the modified American Heart Association classification [25]. Initially, each segment was assessed regarding image quality and evaluability. Segments were considered non-evaluable if artifacts prevented reliable assessment of the lumen or the vessel wall (e. g. due to motion, image noise or heavy calcification). Secondly, each segment was visually evaluated with regard to the presence of plaques and stenoses. Lesions were quantified for stenosis by visual estimation, comparing the minimal lumen of the stenotic area with the lumen of the adjacent proximal unaffected segment, and expressed in terms of diameter stenosis: <50 % (non-obstructive CAD) or ≥50 % (obstructive CAD).

### Coronary artery calcium score

To obtain the CAC score, a non-enhanced scan was performed, using a prospectively ECG-triggered scan protocol: detector configuration 64 x 0.625 mm, rotation time 350 ms, tube potential 120 kV, tube current 200 mA.

The CAC score was calculated using semi-automatic software (SmartScore 4.0, GE Healthcare, Milwaukee, WI, USA) on the Advantage Workstation 4.4 (GE Healthcare, Milwaukee, WI, USA). The total calcium burden of the coronary arteries was reported in terms of AJ-130 score, based on the scoring algorithm of Agatston et al [26].

### Statistical methods and data management

Initially, data were characterized using descriptive statistics and graphically assessed for normal distribution. Continuous variables are presented as mean ± SD or median (range). Since the variables to be compared were not normally distributed, non-parametric tests were used. Hence, statistical comparisons in order to test differences between two arbitrary independent groups were made by use of the Mann–Whitney U-test. The Spearman test was used to test correlation between variables. All analyses were carried out by use of the SAS system (The SAS system for Windows 9.3, SAS Institute Inc., Cary, NC, USA.) and the 5 % level of significance was considered.

A post-hoc power analysis yielded a power of 0.68 to show a 20 percent decrease in RHI in subjects with CAD compared to those without CAD, given the 5 % level of significance.

## Results

All 58 study participants underwent coronary CTA, with subsequent plaque burden analysis, as well as testing for peripheral endothelial function by means of RH-PAT. A non-enhanced cardiac CT scan for CAC scoring was obtained in 51 persons. Ultrasound of the carotid arteries and calculation of IMT was successfully performed in 57 study participants.

Baseline characteristics of the study group are presented in Table 1. Importantly, only three subjects were on lipid-lowering medication. Risk assessment was performed according to the Framingham risk algorithm [1]. Thirty-one participants (53 %) had a 10-year risk of 10 % or less, whilst 18 (31 %) had a 10-20 % risk and 9 (16 %) had an elevated risk exceeding 20 %.

**Table 1:** Characteristics of study group (n = 58)

Age (years)	61 ± 6
Female	39 (67 %)
Present smoking	2 (3 %)
Prior smoking	23 (40 %)
Family history of CAD	14 (24 %)
Diabetes mellitus	0 (0 %)
Treated hypertension	6 (10 %)
Treated hyperlipidemia	3 (5 %)
BMI (kg/m^2^)	25.8 ± 3
SBP	129 ± 13
DBP	80 ± 6
Total cholesterol (mmol/l)	5.6 ± 0.8
LDL (mmol/l)	3.7 ± 0.8
HDL (mmol/l)	1.5 ± 0.4
Triglycerides (mmol/l)	1.0 ± 0.4

Abbreviations: BMI, body mass index; CAD, coronary artery disease; SBP, systolic blood pressure; DBP, diastolic blood pressure; LDL, low-density lipoprotein; HDL, high-density lipoprotein; SD, standard deviation. Data are presented as mean ± SD or absolute value (percentage)

When analyzing the coronary CTA, only 2 % of coronary segments were considered non-evaluable. Coronary CTA showed normal coronary arteries, i e neither obstructive nor non-obstructive CAD, in 25 (43 %) study participants. Thirty-two (55 %) persons had non-obstructive CAD and only one person (2 %) had obstructive CAD. The RHI in the study group ranged from 1.4 to 4.9, with a median of 2.2. Carotid artery IMT ranged from 0.49 mm to 0.99 mm, with a median of 0.70 mm. Even though the main focus of the ultrasound examination was the common carotid artery, there were three subjects with evident plaques in the carotid bulb. All of these subjects had CAD by coronary CTA. The CAC score ranged from 0 to 1882, with a median of 4.

**Fig. 1:**
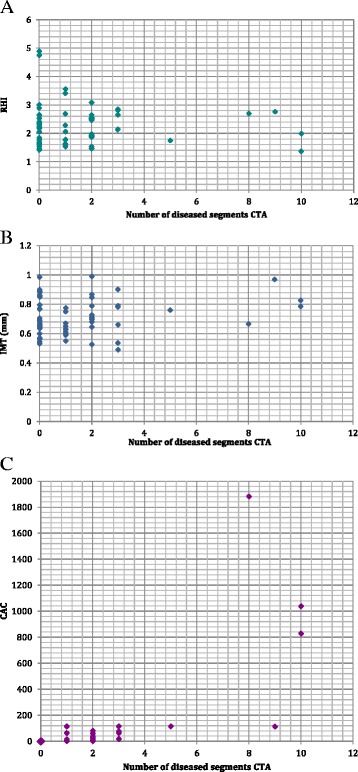
Correlation between number of diseased segments, as determined by coronary CTA, and RHI (**a**), IMT (**b**) and CAC (**c**). The larger point (0, 0) in (**c**) corresponds to 23 subjects. Abbreviations: CTA, computed tomography angiography; RHI, reactive hyperemia index; IMT, intima-media thickness; CAC, coronary artery calcium

When comparing the groups with and without evidence of CAD by coronary CTA, no statistically significant differences were found concerning RHI or IMT. Nor was there any correlation between the number of diseased segments on coronary CTA and RHI (Spearman correlation coefficient 0.13) or IMT (Spearman correlation coefficient 0.098). Not surprisingly, there was a statistically significant difference in CAC scores when comparing the groups with and without CAD demonstrated by coronary CTA (p < 0.0001). Similarly, there was a strong correlation between the CAC score and the number of diseased coronary segments (Spearman correlation coefficient 0.86, p < 0.0001). Results are shown in Table 2 and Fig. 1. Still, among persons with a 0 CAC score 3 (14 %) had evidence of CAD by coronary CTA.

**Table 2:** Comparison between groups with and without CAD (as determined by coronary CTA)

	No CAD n = 25	CAD n = 33	*P*
RHI	2.2 (1.4-4.9)	2.1 (1.4-3.6)	*ns*
IMT (mm)	0.70 (0.53-0.99)	0.71 (0.49-0.99)	*ns*
CAC	0 (0–4)	36 (0–1882)	<0.0001

Abbreviations: CAD, coronary artery disease; CTA, computed tomography angiography; RHI, reactive hyperemia index; IMT, carotid artery intima-media thickness; CAC, coronary artery calcium score; *ns*, non significant. Values are presented as median (range)

## Discussion

The most important findings were that peripheral endothelial function by the RH-PAT method and IMT did not differ significantly between the groups with and without evidence of CAD by coronary CTA, and that there was no correlation between RHI or IMT and number of diseased coronary artery segments. Not surprisingly, the CAC score was significantly higher in the group with CAD compared to the group without, and there was a strong association between CAC and the number of diseased segments. However, a 0 CAC score did not exclude the presence of atherosclerotic plaques on coronary CTA.

To the best of our knowledge, this is the first publication comparing endothelial function by the RH-PAT method with coronary CTA plaque burden. It is one of the few studies comparing IMT with plaque burden, as determined by coronary CTA, and is unusual in studying a mainly female group with a predominantly low to intermediate cardiovascular risk profile.

Matsuzawa et al. studied 140 women with chest pain, who underwent endothelial function testing by RH-PAT as well as conventional coronary angiography and demonstrated a significantly worse endothelial function in patients with obstructive and non-obstructive CAD compared to patients with no CAD [27]. The RHI was markedly worse in the patients with CAD of that study compared to the RHI of the patients with CAD of the present study. This difference might reflect a significantly greater risk factor burden of the patients of the previous study. The fact that the present study analyzed CAD by coronary CTA instead of using conventional coronary angiography might also have affected the results. Since coronary CTA permits visualization of non-stenotic lesions that might not be visible on conventional coronary angiography, more patients with limited disease are likely to end up classified as having CAD when coronary CTA is used instead of conventional coronary angiography. Rohani et al., on the contrary, reported a lack of association between endothelial function, expressed in terms of flow mediated dilation of the brachial artery, and the angiographic extent of coronary stenoses in patients with severe CAD [28]. A possible implication of their findings might be that endothelial function testing is of limited value for patients with pre-existing severe CAD. It has indeed been suggested that endothelial function testing, and in particular testing involving the microvasculature as is the case for the RH-PAT method, might be more suitable for subjects with a low prevalence of risk factors [29]. Our findings for the present study group, however, do not support this hypothesis.

With respect to the lack of association between IMT and the presence and extent of CAD, our findings differ from those of some previous studies [21, 30, 31]. These studies, however, have mainly focused on patient groups with a more pronounced risk profile and with more severe CAD, as determined by cardiac CT or by conventional coronary angiography, compared to the present study group. Our findings are partly supported by a recent study by Schroeder et al., who found no significant relationship between carotid artery ultrasound findings and coronary CTA plaque burden [22]. They did however demonstrate a higher sensitivity of carotid ultrasound compared to CAC for coronary CTA plaque burden. Their study focused on asymtomatic patients referred for risk stratification, with a cardiovascular risk profile and a prevalence of CAD similar to that of the present study. The increasing evidence that IMT of the common carotid artery may not be a good marker for coronary atherosclerosis also supports the results of the present study [14].

The finding of a strong correlation between CAC and the presence and extent of CAD as determined by coronary CTA is not surprising, since the two measures correspond to two aspects of the atherosclerotic process of the coronary vascular bed.

An important limitation of the present study is the limited sample size. Thus, lack of significant differences and correlation may be caused by lack of power to detect such differences. Since no correlations were found, additional statistical analyses to determine sensitivity, specificity and predictive values were not performed. The study group mainly reflects a northern European ethnic group, which might affect generalizability. Since the study group was recruited among clinically healthy subjects, the cardiovascular risk profile was in general favorable and there were no patients with for instance diabetes mellitus or HIV infection. This might also affect generalizability, due to differences in plaque burden in specific patient groups. For instance, a greater proportion of non-calcified plaques was demonstrated in HIV patients compared to healthy subjects [32]. The validity of the results of this study is dependent on the validity of the methods used. Although 64-detector coronary CTA has proven highly sensitive in detecting atherosclerotic plaques, there is a limitation to its spatial resolution, which makes it hazardous to assess very small plaques, in particular if located distally in the coronary arteries [33]. The carotid ultrasound examination focused on the IMT of the common carotid artery, whereas additional analyses such as plaque detection might have yielded different results. Indeed, plaque assessment of the carotid arteries, including the carotid bulb and the internal carotid artery, has been shown to predict CAD and outcome better than IMT alone [14]. Similarly, the choice of RH-PAT, in order to ensure a highly reproducible method for measuring endothelial function, instead of for instance brachial artery flow mediated dilation might also have affected the results. It has been shown that different risk factors affect endothelial function of the microvasculature (as measured with RH-PAT) and the conduit vessels differently [34]. Another factor influencing our results might be the fact that endothelial function was determined with one single test. Repeated measurement has been proposed in order to augment accuracy, since endothelial function is a dynamic process, which can be transiently affected by a variety of factors [29]. Importantly, our study did not examine the prognostic value of RH-PAT or IMT.

The contradictory findings of various studies may in part be related to differences of the study groups, with highly variable prevalences of risk factors and CVD. Another important issue is the choice of methods, as discussed above. One explanation for the lack of association between IMT or endothelial function and CAD demonstrated in this study might be the fact that the different measures reflect the presence of different underlying cardiovascular risk factors, to which the various vascular beds are more or less susceptible. Notably, Bauer et al. demonstrated that certain risk factors have different impacts on IMT and CAC, with diabetes having a relatively higher impact on IMT and blood pressure showing the strongest association with CAC [35]. In some studies endothelial dysfunction has been shown to precede the appearance of CAD or to predict the progression of IMT, hence suggesting that it represents an earlier step in the atherosclerotic process [36, 37]. Our results, however, do not entirely support this hypothesis, since a large number of subjects with CAD evident by coronary CTA displayed a normal endothelial function.

## Conclusions

Our results suggest that neither measurement of peripheral endothelial function by RH-PAT nor carotid ultrasound assessment of IMT alone can reliably be used to predict subclinical coronary atherosclerosis, as determined by coronary CTA.
